# Anion Exchange Membranes with 1D, 2D and 3D Fillers: A Review

**DOI:** 10.3390/polym13223887

**Published:** 2021-11-10

**Authors:** Riccardo Narducci, Emanuela Sgreccia, Philippe Knauth, Maria Luisa Di Vona

**Affiliations:** 1 Department Industrial Engineering and International Laboratory “Ionomer Materials for Energy”, University of Rome Tor Vergata, I-00133 Rome, Italy; emanuela.sgreccia@uniroma2.it (E.S.); divona@uniroma2.it (M.L.D.V.); 2 CNRS, Madirel (UMR 7246) and International Laboratory “Ionomer Materials for Energy”, Aix Marseille University, F-13013 Marseille, France; philippe.knauth@univ-amu.fr

**Keywords:** AEMFCs, carbon nanotubes, LDH, graphene oxide, silica, zirconia, MOF, carbon dots

## Abstract

Hydroxide exchange membrane fuel cells (AEMFC) are clean energy conversion devices that are an attractive alternative to the more common proton exchange membrane fuel cells (PEMFCs), because they present, among others, the advantage of not using noble metals like platinum as catalysts for the oxygen reduction reaction. The interest in this technology has increased exponentially over the recent years. Unfortunately, the low durability of anion exchange membranes (AEM) in basic conditions limits their use on a large scale. We present in this review composite AEM with one-dimensional, two-dimensional and three-dimensional fillers, an approach commonly used to enhance the fuel cell performance and stability. The most important filler types, which are discussed in this review, are carbon and titanate nanotubes, graphene and graphene oxide, layered double hydroxides, silica and zirconia nanoparticles. The functionalization of the fillers is the most important key to successful property improvement. The recent progress of mechanical properties, ionic conductivity and FC performances of composite AEM is critically reviewed.

## 1. Introduction

**Figure 1:**
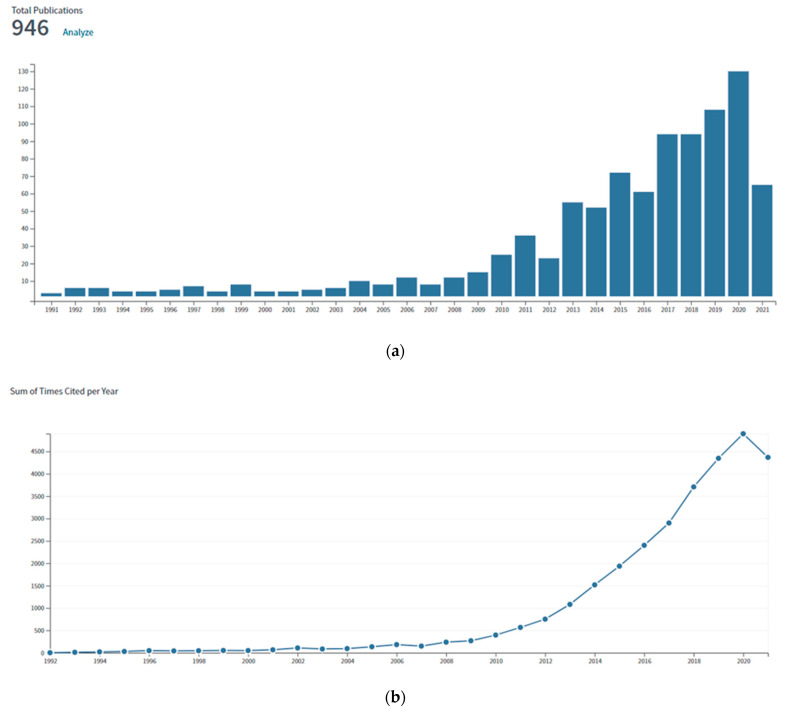
(**a**) Number of publications and (**b**) number of citations on the topic “composite anion exchange membranes” (from ISI Web of Science 2021).

The anion exchange membranes (AEM) are the central element of many technologically relevant devices [1,2,3], first and foremost alkaline membrane fuel cells (FC) [4,5,6]. These fuel cells can significantly reduce the amount of noble metal catalysts for the oxygen reduction reaction (ORR) and may represent the future of FC development. However, although AEM may revolutionize future fuel cell technology, currently they lack several properties that are paramount for the viability of this technology, including long-term stability in the alkaline medium that impacts their durability in operating conditions [7,8,9,10]. One favoured way to improve the properties of ionomeric and more generally polymeric membranes is the development of composite materials, especially using inorganic fillers that are supposed to enhance the mechanical properties and the FC performance. The reduction in ionic conductivity consecutive to the addition of an inert second phase can be mitigated by fillers that present an intrinsic ionic conductivity or by space charge effects near the interfaces between ionomer and inorganic nanoparticles. Furthermore, the composite material may in certain cases show higher long-term stability in alkaline conditions by synergistic effects, due, e.g., to a physical cross-linking (such as acid–base interactions) or a partial crystallization of the polymer near the inorganic phase. Although the application of nanoparticle fillers is promising, their practical investigation started in earnest only a decade ago, as shown in the number of publications and citations on the topic “composite anion exchange membranes” (Figure 1). No comprehensive and critical review exists on this topic, although some related topics have been addressed in the last years [11,12,13,14,15]. 

This review intends thus to summarize the main findings reported in the literature since 2010, including AEM for use in fuel cells, but also in redox flow batteries and water purification applications. We chose a simple and coherent presentation of this wide field by subdividing the literature according to the dimensionality of the fillers. We start from 1D materials, essentially nanotubular solids, including titanate and carbon nanotubes (NTs). Titanate NT exhibit high chemical stability, high specific surface area and good performance in alkaline environments and their aspect ratio can increase the mechanical properties. Carbon NT are also characterized by extreme strength and flexibility so that an increase in mechanical properties is expected. Organic functionalization is the key to obtaining high ionic conductivity. The following chapter summarizes 2D materials with a large part on graphene and graphene oxide (GO). Graphene and GO are mechanically extremely resistant and at the same time present great flexibility. Composites, especially with functionalized graphene and/or reduced GO, are expected to have improved FC performance and conductivity thanks to a better filler distribution and sometimes with the help of ionic liquids. Layered double hydroxides (LDHs) can mitigate the loss of hydroxide ion conductivity, being themselves anion conducting materials. They can also improve the mechanical properties even at 100% RH. The final part discusses 3D fillers, with a large section on SiO_2_ and silicates, as well as other metal oxides including alumina, titania and zirconia nanoparticles as well as metal–organic frameworks (MOFs). The organic functionalization of the oxides plays a decisive role in increasing the performance of composite materials. The first type of polymers used as matrix are inert, non-ionic, used as an absorbent for alkaline solutions, including synthetic poly(vinyl alcohol) with the general formula [-CH_2_CH(OH)-]n and chitosan, an aminated polysaccharide, composed of N-acetyl-D-glucosamine and D-glucosamine linked by β(1-4) bonds. The second type of matrix are ionomers that present an intrinsic ionic conductivity. They include fluorinated and hydrocarbon polymers, especially commercial, inexpensive aromatic polymers such as poly (2,6-dimethyl-1,4-phenylene oxide) (PPO) and polysulfone (PSU). Both are thermoplastics, known for their toughness, resistance at high temperature and especially PPO for alkaline stability. 

We cover in this review particularly ionic conductivity, mechanical properties and device performance, especially in fuel cells. Several tables in the manuscript summarize information on the reviewed AEM.

## 2. 1D Materials

### 2.1. Titanate (TNTs) and Halloysite Nanotubes (HNTs)

Titanate nanotubes (TNTs) were first described in the 1990s. These nanostructured tubular materials present excellent characteristics and performances [16]. Among them, titanium dioxide TiO_2_ NT exhibit high chemical stability and catalytic activity, a high specific surface area, strong metal–support interaction, and good performances in alkaline and acidic environments. TiO_2_ NT have a one-dimensional (1D) structure and exists as bundled tubes with a length between 1 and 220 µm and a diameter between 30 and 80 nm. The morphology of TNT depends on which synthesis method was used, including electrochemical anodization [17,18], a template-assisted method, sol–gel chemistry [19], etc. given all these characteristics, among other applications, TNT are used as filler in fuel cell membranes [16].

One of the earliest examples of composite AEM with TNT was presented in 2018 by Elumalai and Sangeetha [20]. The same authors incorporated TNT covalently linked with an imidazolium-based ionic liquid (IL) into quaternized polysulfone (QPSU) with triethylamine (TEA) [21]. The presence of the IL increased the IEC; the best conductivity was observed for 5 wt% IL-TNT (21 mS cm^−1^, 30 °C), due to the uniform distribution of the filler without any cluster formation. The same composite membrane reached a maximum tensile strength (TS) of 43 MPa and a fuel cell power density of 302 mW cm^−2^ at 60 °C under 80% RH. Quaternized TNT (QTNT) were also incorporated in quaternized poly(arylene ether ketone) (QPAEK) [22]. 

**Figure 2:**
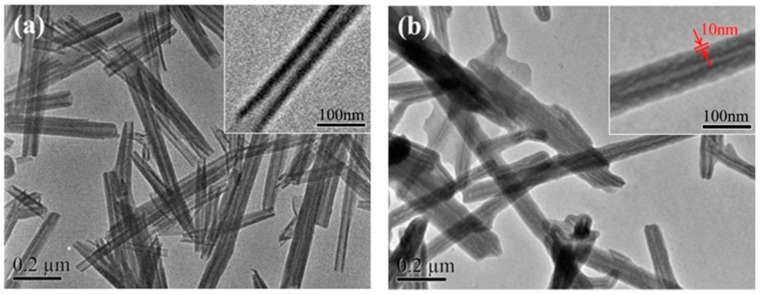
TEM images of (**a**) halloysite nanotubes and (**b**) quaternized halloysite nanotubes. Reproduced with permission from Ref. [23].

Halloysite nanotubes (HNTs), natural two-layered aluminosilicate clay minerals, were quaternized (QHNTs) with imidazolium groups bearing different functionalities (butyl, decyl, carbethoxyl, and benzyl moieties) and then embedded into a chitosan (CS) matrix [23]. Figure 2 shows the TEM images of HNTs and QHNTs. HNTs presented a clear tubular structure while after modification QHNTs showed a well-defined polymeric layer. 

The incorporation of 5 wt% of HNTs elevated the TS and Young’s modulus (E) to 52 MPa and 1100 MPa, respectively. The membranes containing QHNTs functionalized with carbethoxyl groups showed the best hydroxide conductivity under hydrated conditions: with 7.5 wt% the conductivity reached 17 mS cm^−1^ at 90 °C, which is still modest. 

### 2.2. Carbon Nanotubes (CNTs)

Carbon nanotubes are a relaxation of the spherical structure of fullerene rolled up on itself, obtaining the typical cylindrical structure. They can be divided into two types:

(a) SWCNT (Single-Walled Carbon NanoTubes) consist of a single graphitic sheet wound on itself.
(b) MWCNT (Multi-Walled Carbon NanoTubes) are formed by several sheets coaxially wound one on top of the other.

Very promising in their electrical properties, they are also characterized by extreme strength and flexibility, making them suitable for use as reinforcing fibers in composites. Several authors incorporated functionalized multi-walled carbon nanotubes (MWCNTs) into various types of AEM. 

#### 2.2.1. Poly(vinyl alcohol) (PVA)

**Figure 3:**
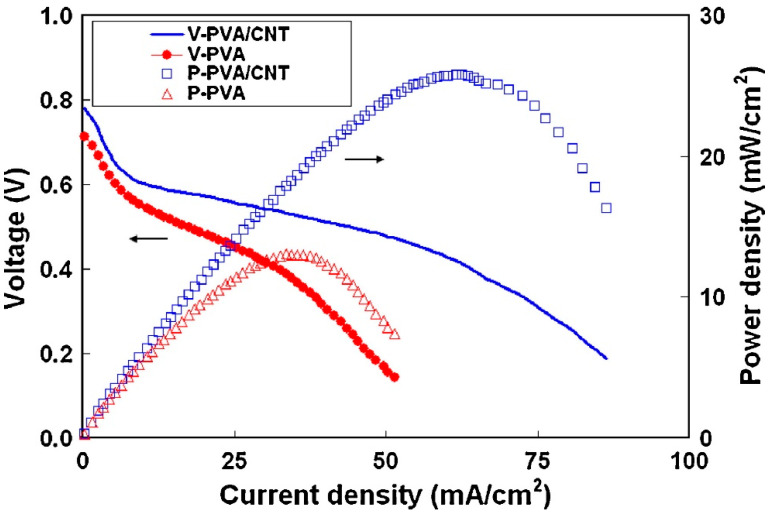
Effect of MWCNT addition in PVA on DMAFC performance at 30 °C (anode: 2 M MeOH in 6 M KOH, flow rate of 5 mL min^−1^; cathode: humidified O_2_, flow rate of 100 mL min^−1^). Reproduced with permission from Ref. [24].

Poly(vinyl alcohol) (PVA) is an inert polymer, soluble in warm water, that acquires ion conductivity through the addition of KOH or other alkaline solutions. One of the first examples of composite with PVA and MWCNT was reported by Pan et al. in 2011 and applied in direct methanol alkaline fuel cells (DMAFC). The methanol permeability decreased from ~3.6 × 10^−7^ cm^2^ s^−1^ for pristine PVA to 3.0 × 10^−7^ cm^2^ s^−1^ for composite membranes. The conductivity increased with the presence of MWCNT and was reported between 52 and 118 mS cm^−1^ (in 2 M and 6 M KOH at 30–60 °C). Figure 3 presents the DMAFC performance and the improvement due to the incorporation of CNTs [24].

PVA-functionalized MWCNTs were also prepared using an ozone-mediated method [25]. The MWCNTs were added to PVA solution and stirred at 80 °C for 3 h to graft the PVA polymer onto the MWCNT. The dry PVA/MWCNT films were then immersed in 1–8 M KOH solutions. PVA containing 0.05% of functionalized MWCNT exhibited the maximum power density of 39 mW cm^−2^ obtained with 2 M MeOH and 6 M KOH at 60 °C, consistent with the previous work. 

Functionalized MWCNTs with pendant Fe_3_O_4_ nanoparticles (FeCNT) were incorporated into a PVA film [26]. High resolution HRTEM images showed a diameter of the MWCNT of 20−30 nm with Fe_3_O_4_ nanoparticles on the MWCNT surface. For a PVA–0.15 FeCNT sample an open-circuit potential of 0.87 V and a maximum power density of 88 mW cm^−2^ were obtained in DMAFC (2 M MeOH in 6 M KOH at 60 °C). To improve the chemical compatibility between CNTs and PVA, the CNTs were functionalized with PVA chains [27]. The maximum power density for 3 M EtOH in 5 M KOH solution was 65 mW cm^−2^ for the composite and 31 mW cm^−2^ for pristine PVA film. 

Membranes composed of a semi-interpenetrating network structure of cross-linked PVA, poly-diallyldimethylammonium chloride (PDDA) and hydroxylated MWCNTs-OH were tested in AEMFC [28]. The cross-link was obtained by thermal treatment of PVA and subsequent immersion in acid solution with 10 wt% glutaraldehyde (GA). The tensile stress at break of PVA/PDDA membranes with 3 wt% MWCNTs-OH was 40 MPa, the elongation at break (%) and Young’s modulus were 12% and 783 MPa, respectively. The maximum OH^−^ conductivity was 30 mS cm^−1^ at RT with an improved alkaline stability. The (PVA/PDDA/1 wt% MWCNTs-OH) MEAs in an AEMFC with 0.5 mg Pt cm^−2^ at the anode and cathode side attained a power density of 66 mW cm^−2^ at 40 °C; using 3.2 mg cm^−2^ of cobalt phthalocyanine at the cathode side, the power density decreased to 14.0 mW cm^−2^. 

#### 2.2.2. Chitosan (CS)

Only a few examples of composites containing chitosan and CNTs are present in the literature. CS was used as a matrix for MWCNTs-OH and ionic liquids quaternized with isoquinoline moieties bearing ammonium groups [29]. Quaternized chitosan (QCS) and functionalized CNTs were prepared with the aim to improve the mechanical properties [30]. CNTs were also functionalized with quaternized silica obtained by in situ sol–gel methods [31]. The incorporation of 5 wt% of functionalized CNTs enhanced the conductivity to 43 mS cm^−1^ at 80 °C, about two times higher than bare QCS. DMAFC tests with composite membranes produced a maximum power density at 60 °C of 81 mW cm^−2^, higher than pure QCS (52 mW cm^−2^). 

#### 2.2.3. Aromatic Polymers 

Except in one case [32], the imidazolium group (Im) was the preferred choice in all composites containing aromatic polymers.

Jin and Bai in 2013 blended methyl-Im quaternized PPO (PPO–MIm) with MWCNTs functionalized with a polymeric ionic liquid containing Im moieties (PIL(BF4)–MWCNTs) [33]. The conductivity increased in the presence of the filler (95% more with respect to PPO–MIm) together with an enhancement in TS up to 13 MPa. 

**Figure 4:**
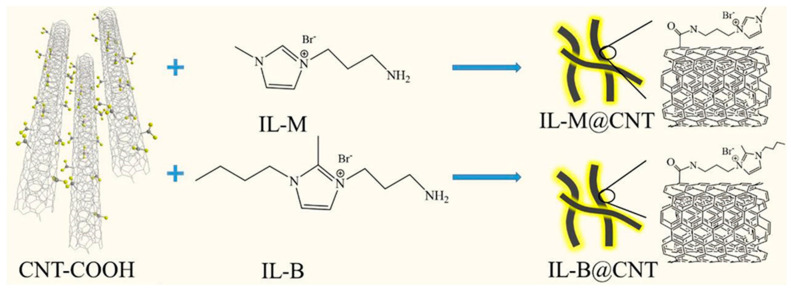
Synthesis of ionic liquids functionalized CNTs (IL-(M;B)@CNT). Reproduced with permission from Ref. [34].

Imidazolium ionic liquids (ImILs) modified carbon nanotubes (IL@CNT) were also inserted into imidazolium-modified poly(ether ether ketone) (Im-PEEK) [34]. Two types of ionic liquids (IL-M and IL-B) with different alkaline stability and chain length were chemically attached to CNTs (Figure 4). The authors stated that the introduction of IL@CNT provided additional ion hopping positions and 1D long range ion-conducting channels.

The composite containing IL-B@CNT showed a higher conductivity (135 mS cm^−1^ at 70 °C and 100% RH) confirming the beneficial effect of the long chain (pure Im-PEEK 80 mS cm^−1^), while the residual hydroxide conductivity (Im-PEEK/IL-B@CNT-8, after 48 h in 2 M KOH at 50 °C) was 77%. The peak power density in AEMFC was 81 mW cm^−2^ at 50 °C [34]. 

Im-PEEK was also used as a hosting matrix for poly(vinyl imidazole) functionalized carbon nanotubes (PVI@CNT) [35]. The imidazolium groups of the filler reacted with the Im-PEEK to form a cross-linking structure along the nanotubes. The nano-hybrid membrane with 15 wt% of the filler (Im-PEEK/PVI@CNT-15) showed a conductivity of 121 mS cm^−1^ at 70 °C and 100% RH. According to the authors, the improvement of the hydroxide conductivity was due to additional anion transport pathways built along the interface between the PVI@CNT and the polymer matrix, with an increase in the connectivity and the ion transport. The maximum power density in a single H_2_/O_2_ fuel cell was 129 mW cm^−2^ at 60 °C under 100% RH. 

Via co-electrospinning, an imidazolium functionalized MWCNTs was incorporated into imidazolium functionalized polysulfone (Im-PSU) [36]. In fully hydrated conditions, the tensile stress increased from 5.6 MPa for a cast Im-PSU to 24.4 MPa for electrospun ImPSU with 0.2 wt% of MWCNTs. The maximum value of OH-conductivity was obtained for 0.4 wt% of FMWCNT with 100 mS cm^−1^ at 60 °C, but the residual conductivity after immersion in 1 M NaOH at 60 °C for 168 h was 62%. 

Table 1 summarizes information on the various composites, including polymer and 1D filler type, remarks and references.

We have seen in this section different 1D nanofillers such as CNT, halloysite and titanate NT, commonly used for the fabrication of hybrid materials. One-dimensional tubular and well designed nanofillers can provide ion conducting groups, construct ion conducting pathways along the interphase region and improve the anti-swelling property of the nanohybrid membrane. For these reasons, as shown previously, both the mechanical properties and the residual conductivities after the alkaline test increase, and above all the performances in FC show an improvement compared to the bare polymers.

## 3. 2D Materials

### 3.1. Layered Double Hydroxides (LDHs)

Layered double hydroxides (LDHs) are inorganic lamellar ionic materials belonging to the group of anionic clays and their synthesis has a low cost. The structure of LDH is based on Mg(OH)_2_ brucite-type blocks where the replacement of M^2+^ with M^3+^ cations gives positively charged layers, balanced by mobile anions in the interlayer, which can be reversibly inserted. The lamellae are linked by Van der Waals forces. LDHs with an acceptable anionic conductivity and excellent stability in alkaline media can be used successfully as filler in AEM [37]. Well dispersed LDHs increase the mechanical properties of the matrix and can help to mitigate the loss of conductivity observed in the case of non-conducting fillers. 

#### 3.1.1. Poly(vinyl alcohol) (PVA) and Chitosan (CS)

One of the first attempts to use LDH in a composite date back to 2012 when Zhao et al. dispersed LDH in crosslinked PVA [38]. SEM images showed a good homogeneity without aggregates or chunks until 30 wt% of LDH (30LDH). FC with a PVA/20LDH membrane achieved a maximum power density of 82 mW cm^−2^ at 80 °C. 

Exfoliated LDH nanosheets, obtained via the filtration process, were inserted in quaternized PVA [39]. The conductivity of the LDH(NO_3_^−^) membrane after exfoliation and restacking was around 200 mS cm^−1^ at 80 °C, which seems surprisingly high especially for nitrate ions and also higher than the OH^−^ form. The improvement of conductivity was attributed to the exposed surfaces of the nanosheets, the enhanced water uptake, and long-range ordered ionic channels. For the composite with 10 wt% of PVA, (LDH(NO_3_^−^)/QPVA-10) was reached at the same temperature 172.1 mS cm^−1^. 

**Figure 5:**
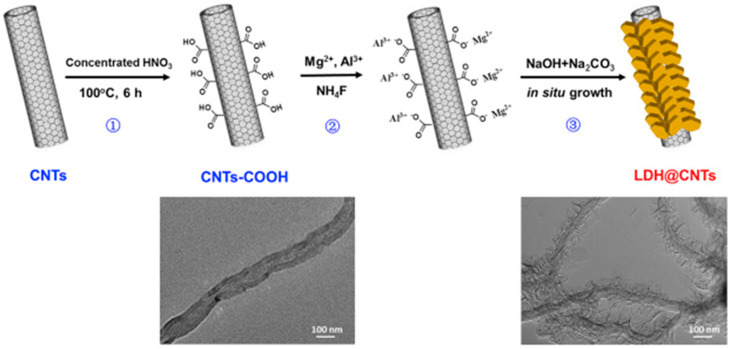
Preparation of LDH coated carbon nanotubes (LDH@CNTs). Reproduced with permission from Ref. [42].

Other examples concern the use of mixed membranes composed of QCS and PVA; they hosted LDH intercalated with glycine betaine [40], an LDH flower-like hierarchical structure wrapped on SiO_2_ nanospheres (LDH@SiO_2_) [41], and carbon nanotubes coated with LDH (LDH@CNTs) [42]. In the last example, LDH nanosheets were anchored on carbon nanotubes by a NH_4_F-assisted in situ coating method (Figure 5) [42].

The conductivity of the composite membrane was 47 mS cm^−1^ at 80 °C, in comparison with 29 mS cm^−1^ for the pristine membrane. With the increase in the content of LDH@CNTs the alkaline stability improved and QCS/PVA-4%-LDH@CNTs membrane showed a residual ionic conductivity of about 73% after 192 h in 1 M KOH at 40 °C. The performance with 1 wt% of filler in a DMAFC (2 M MeOH, 5 M KOH) at 80 °C gave a maximum power density of 107 mW cm^−2^ [42]. 

#### 3.1.2. Polysulfone (PSU)

**Figure 6:**
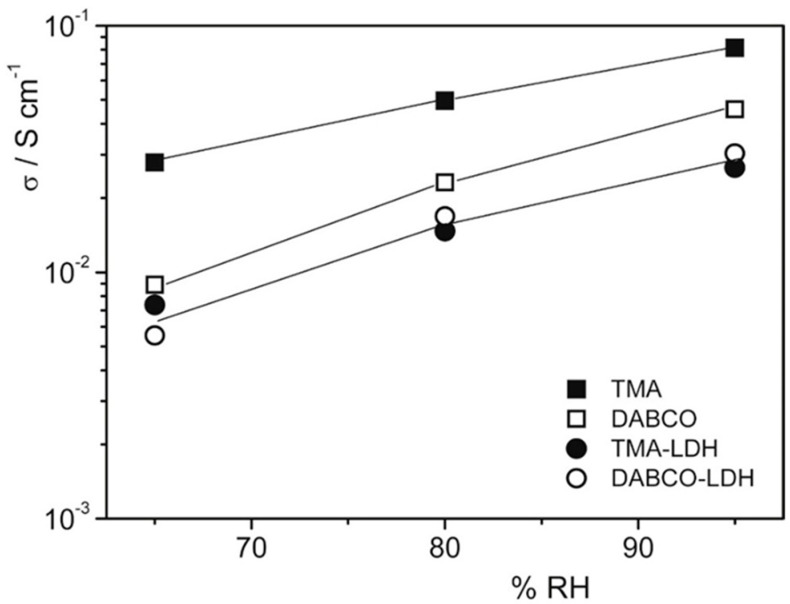
Conductivity measurements at 60 °C as a function of RH% of membranes after treatment at 25 °C in 2 M KOH for 24 h. Reproduced with permission from Ref. [37].

An attempt to use an inexpensive commercial polymer was proposed in 2017 by Di Vona et al. [37]. The composites were prepared from QPSU grafted with TMA or 1,4-diazabicyclo[2.2.2]octane (DABCO) and 14 wt% of LDH, synthesized by the urea method with a composition Mg_0.62_Al_0.38_(OH)_2_(Cl)_0.38_·0.6H_2_O. The ionic conductivity at 60 °C as a function of RH is reported in Figure 6: composite membranes showed lower conductivity values due to the lower water uptake, while polymers with TMA were more conductive because of the higher water content. The mechanical properties in fully humidified conditions were improved, with a three-fold increase in Young’s modulus for composites (~600 MPa vs. ~200 MPa). The membranes were treated in alkaline conditions at 60 °C without losing their properties. 

Meanwhile, Zhang prepared LDH by the urea method, which was incorporated from 3 to 10 wt% into TMA-PSU membranes [43]. The 5 wt% LDH nanocomposite membrane showed the highest TS (21 MPa), the lowest elongation at break (11%), and the highest ionic conductivity (24 mS cm^−1^ at 60 °C). Pizzoferrato et al. proposed a composite membrane based on PSU-DABCO and ZnAl-LDH containing an ionic liquid (1-butyl-3-methylimidazolium hydrogen sulfate, BmimSO_4_) [44]. This strategy enabled the intercalation of IL into the interlamellar space of LDH with the aim of enhanced ionic conductivity. In fact, at 100% RH and 25 °C, the membranes with 7 wt% of filler reached a conductivity of 25 mS cm^−1^ in OH^−^ form and maintained 16 mS cm^−1^ after alkaline treatment in 2 M KOH at 60 °C for 24 h [44]. 

#### 3.1.3. Poly(phenylene oxide) (PPO)

**Figure 7:**
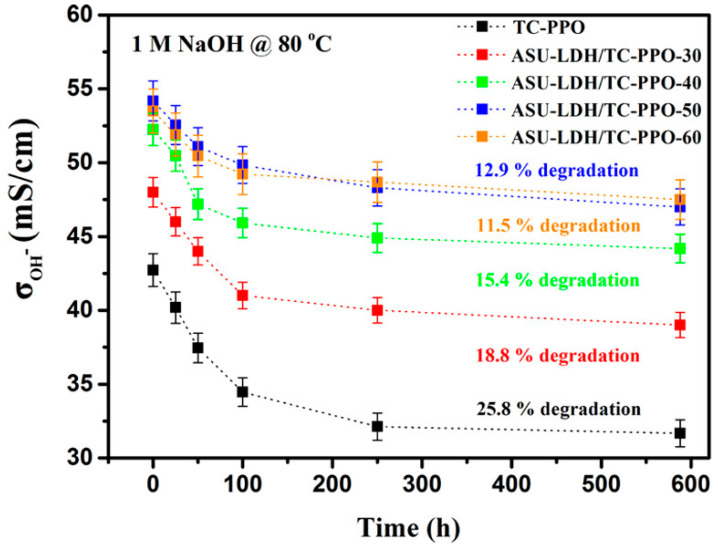
Hydroxide conductivity variations vs. time of TC-PPO and hybrid membranes with ASU-LDH at 30 °C. Reprinted with permission from Ref. [45]. Copyright (2018) American Chemical Society.

A porous-sandwich structure was synthesized by electrostatic spraying of ammonium layered double hydroxide (QLDH) on the surface of triple-cation side chains of PPO (TC-PPO) [45,46]. The composite membranes with higher IEC values (from 3.12 to 3.39 meq g^−1^), due to the presence of ammonium groups in QLDH layers, showed a high hydroxide conductivity (122 mS cm^−1^ at 80 °C). The FC performance at 60 °C achieved a maximum power density of 267 mW cm^−2^ at a current density of 554 mA cm^−2^, values higher than those of membranes without QLDH but still far from those recorded for from Nafion 112 [46]. The same authors synthetized LDH functionalized with 3-hydroxy-6-azaspiro [5.5] undecane cations (OH-ASU) to increase the ion exchange capacity [45]. The ASU-LDH filler was combined with TC-PPO to fabricate three-decker ASU-LDH/TC-PPO hybrids with IEC up to 3.90 meq g^−1^. The OH^−^ conductivity, shown in Figure 7, increased from 79 mS cm^−1^ for TC-PPO to 112 mS cm^−1^ for ASU-LDH/TC-PPO-50. After stability tests in 1 M NaOH at 80 °C for 588 h, only an 11.5% drop in OH^−^ conductivity was observed.

An electric field was sometimes applied to induce the formation of ion channels along the through-plane direction [47,48]. Aligned LDH functionalized with N-spirocyclic ammonium groups and PPO showed a maximum ion conductivity of 110 mS cm^−1^ at 80 °C and an enhanced alkali resistance with 83% residual conductivity [48]. The good stability was attributed to the formation of electric field-induced ion channels that reduced the local alkaline concentration near quaternized groups and decreased the possibility of the hydroxide to attack ammonium groups. 

Composite membranes with DABCO-PPO and 30 wt% MgAl-LDH were prepared in 2020 by Pasquini et al. [49]. The high amount of the second phase was chosen in order to understand the effect of the inorganic filler on alkaline and hydrolytic degradation. The stability behaviour, studied by different techniques, was comparable to the initial ionomer showing a fast degradation in the first hours.

#### 3.1.4. Poly(vinylidene fluoride) (PVDF)

Only one example was reported in the literature concerning PVDF: a bioinspired geometrical templating of PVDF substrate containing electrospun MgAl-LDH for solid-state AFC [50]. The hybrid substrate was morphologically similar to the coconut flower (Cocosnucifera). Efficient pore-filling by LDH was demonstrated by SEM and the inorganic phase enhanced TS two-fold (14 MPa). The Cl^−^ conductivity at 80 °C was 92 mS cm^−1^ at 100% RH and 18 mS cm^−1^ at 50% RH. 

### 3.2. MXenes

MXenes are a family of 2D layered materials which include transition metal nitrides, carbides, and carbonitrides produced by selective exfoliation of MAX phases, where “M” refers to early d-block transition metals, “A” to groups 13 and 14 (main sp elements) and “X” to carbon or nitrogen atoms [51]. MXenes present several properties, such as hydrophilicity, fast ion transport and intercalation, large surface area, etc., provoking interest in many scientific applications including FC. 

By solution blending method two kinds of 2D nanomaterial Ti_3_C_2_T_x_ (T_x_ stands for the hydrophilic surface terminations-O, -OH, -F, etc.) were incorporated into QPSU [52]. The mechanical strength and the conductivity were slightly increased adding 3 wt% of LiF-Ti_3_C_2_T_x_ and NH_4_HF_2_-Ti_3_C_2_T_x_. The maximum power density in a single cell at 60 °C with H_2_/O_2_ atmospheric pressure and Pt loading of 0.4 mg cm^−2^ in both sides for QPSU/LiF-Ti_3_C_2_T_x_ and QPSU/NH_4_HF_2_-Ti_3_C_2_T_x_ attained 74 mW cm^−2^ and 101 mW cm^−2^, respectively. In 2018, Wang et al. dispersed imidazolium functionalized MXene in a CS matrix [53]. The pure CS membrane had a TS of 28 MPa, after the addition of QMXene-NH_2_, the TS improved significantly up to 41 MPa. The conductivity of AEM with 7.5 wt% of QMXene-NH_2_ at 100% RH increased from 1.5 to 4 mS cm^−1^ [53]. 

The AEM composites with 2D fillers are summarized in Table 2, including the type of polymer and 2D filler, remarks and references.

### 3.3. Graphene Oxide (GO) and Graphene 

A very large amount of work and effort was devoted to composite AEM containing graphene oxide (GO) or graphene. The main features for graphene are an extreme mechanical resistance and great flexibility, for GO its hydrophilicity and dispersibility in water, due to epoxy and hydroxyl groups on the basal planes, and carboxyl and carbonyl groups at the edges of layers. In particular, GO was functionalized with many groups creating original and sometimes complex structures.

#### 3.3.1. Poly(vinyl alcohol) (PVA)

PVA-based matrices were often used to host different carbon materials such as exfoliated graphene nanosheets [54], or graphene and sulfonated graphene nanoparticles (PVA/chitosan blend membranes) [55]. Cross-linked PVA and quaternized polyethyleneimine (QPEI) hosted GO functionalized with silica by epoxide ring-opening reactions using APTEOS [56]. Cross-linked composites composed of PVA, quaternized with glycidyltrimethylammonium chloride (GTMAC), and GO (QPVA/GO) was prepared to decrease the EtOH permeability [57]. 

Magnetite nanoparticles on GO were distributed along the through-plane direction in a QPVA matrix by applying an external magnetic field (MF) during the film drying step [58]. The QPVA/0.1%Fe_3_O_4_@GO nanocomposites presented the highest ionic conductivity with 55 mS cm^−1^ and a maximum power density of 200 mW cm^−2^ at 60 °C and 2 M MeOH [59].

#### 3.3.2. Polysulfone (PSU)

Many functionalized graphene or graphene oxide-based composites contained PSU or QPSU as a host matrix [60,61,62,63,64,65,66]. 

TEM images of quaternized polymer brush-functionalized graphene (QPbGs) into QPSU showed a uniform distribution with the amount of QPbGs below 1 wt%. The HCO_3_^−^ conductivity was 56 mS cm^−1^ at 80 °C with 1 wt% of filler [67]. Reduced GO (rGO) functionalized with polydopamine (PDArGO) and QPSU quaternized with 3-(dimethylamino)-1-propylamine reached a hydroxide conductivity of 61 mS cm^−1^ at 80 °C (QPSU-1.5%-PDArGO) [68]. The Young’s modulus raised from 868 MPa for QPSU to 1843 MPa, and the WU increased from 18% to 30% at 80 °C. 

TMA-PSU was cross-linked (XL-QPSU) by rGO modified with short- and long-chain tertiary amines (SrGO and LrGO) [69]. Membranes with elastic long-chain (LrGO) displayed higher ion conductivity due to major nanophase separated morphology. The XL-QPSU-2%-LrGO OH^−^ conductivity was 76 mS cm^−1^ at RT, with respect to a conductivity of 49 mS cm^−1^ for the pristine AEM. The same authors modified rGO with pyrene-containing tertiary amines (TrGO) and polymers (PrGO) via π–π interactions [70]. The functionalized rGO was used to cross-link TMA-PSU. The XL-QPSU-2%-PrGO reached a hydroxide conductivity at 80 °C of 118 mS cm^−1^ and after alkaline treatment in 1 M NaOH for 500 h at 60 °C, the residual conductivity was 88% higher than un-crosslinked QPSU.

Composite AEMs were also expected to reduce the vanadium permeability in vanadium redox flow batteries (VRFB), due to the Donnan exclusion effect. A long alkyl chain (C16) grafted to quaternized GO was bonded to DABCO-PSU and the resulting composites used in VRFB [71]. The long alkyl chain increased the hydrophilic–hydrophobic nanophase separation. The selectivity, assessed by a ratio of ion conductivity and vanadium ion permeability, for 5 wt% of the modified GO, was 19 × 10^5^ S min cm^−3^ and the Coulombic efficiency (CE), was 98% at 60 mA cm^−2^. The good results were attributed to the high ionic conductivity and the extremely low vanadium cross-over. 

#### 3.3.3. Poly(phenylene oxide) (PPO)

Although PPO is widely employed as a polymer in AEMs, only a few examples concern its use in composites with GO. 

Brominated PPO and polyethyleneimine (PEI)-modified GO was proposed by Kulshrestha et al. [72]. A blend of GO (QGO) and cellulose (QCel) quaternized with DABCO, and subsequently XL with DABCO-PPO, displayed an OH^−^ conductivity of 114 mS cm^−1^ at 25 °C [73]. The stability decreased with the increase in QCel amount, due to its oxygenated functionalities susceptible for the nucleophilic attack, and increased with the content of QGO, very stable in alkaline conditions. 

An imidazolium-functionalized PPO (Im-PPO) and an ionic liquid-functionalized GO were used to prepare composite membranes with a hydroxide conductivity of 79 mS cm^−1^ at 80 °C [74]. The alkaline test in 2 M NaOH at 80 °C over 480 h showed a decrease in IEC from 1.90 to 1.34 meq g^−1^. FC tests at 60 °C for 0.5 wt% of IL-GO composite presented a maximum power density of 136 mW cm^−2^ with a current density of 300 mA cm^−2^ under 100% RH. 

Recently, commercial membranes based on PPO (Fumion^®^ FAA) were filled with different amounts of commercial graphene with a surface area of 500 m^2^/g [75]. The SEM morphology showed a uniform graphene inclusion into the polymer matrix; no crack formation and increased roughness were found. The maximum value of OH^−^ conductivity was reached for the sample containing 50 mg of graphene and was 113 mS cm^−1^ in 0.01 M KOH at 80 °C.

Another commercial membrane (JAM-II-07, Yanrun, China) was modified with sulfonated reduced graphene oxide (S-rGO) nanosheets and used in the electrodialysis process [76]. 

#### 3.3.4. Polybenzimidazole (PBI)

Polybenzimidazole (PBI) is a well-known matrix in proton exchange membranes due to its performance at high temperatures; in AEM, PBI can be used after alkaline doping. To overcome the barrier due to the release of alkaline dopants in operative conditions, various strategies were used with the support of GO or rGO.

**Figure 8:**
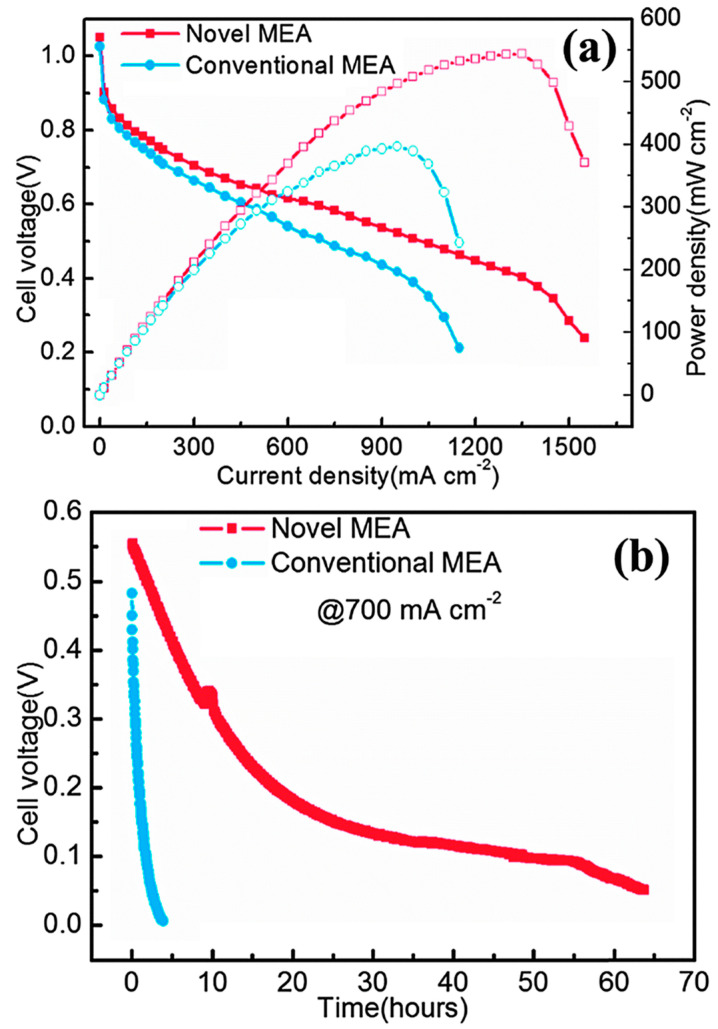
Comparison between conventional and novel MEAs. (**a**) Polarization and power–density curves; (**b**) constant current discharge curves (O_2_, current density 700 mA cm^−2^). Reproduced from Ref. [77] with permission from The Royal Society of Chemistry.

Zeng et al. proposed a MEA formed by a porous PBI membrane (sp-PBI) and a composite as binder formed by PBI and rGO on which the electrocatalyst was deposited [77]. The MEA reached a maximum power density of 544 mW cm^−2^, higher than conventional MEA (397 mW cm^−2^), as shown in Figure 8.

GO nanosheets were coated onto a PBI surface using a spin coater. The thickness of spin-coated with 2 wt% GO was 1–2 μm [78]. The Young’s modulus was improved to 1040 MPa and the tensile strength reached 50 MPa, effects attributed to hydrogen bonds between the edges of GO nanosheets. In DMFC the maximum power density at 80 °C reached 200 mW cm^−2^ with Pt-based catalysts [78]. Two types of GO nanosheets were prepared by microwaves (MGO) and by the modified Hummer’s method (NGO) [79]. MGO was less hydrophilic and more thermally resistant due to it having fewer ether groups and more sp^3^ C–C bonds. The PBI composite with 1 wt% of filler showed an increased conductivity by 38% for NGO and 29% for MGO with respect to pure PBI. Voc and Pmax for NGO were 0.7 V and 310 mW cm^−2^, respectively. 

#### 3.3.5. Other Aromatic Polymers

Quaternized PAEK was filled with different amounts of rGO [80] while poly(phthalazinone ether ketone) (PPEK) hosted cyclodextrins modified with trimethylammonium groups on GO (QA-CDβ@GO) [81]. Imidazolium-functionalized Im-GO inserted into Im-PEEK showed a tensile strength of 36 MPa, 37% higher than pristine Im-PEEK [82]. The conductivity at 70 °C and 100% RH reached 140 mS cm^−1^. The hybrid showed in H_2_/O_2_ FC tests a power density of 50 mW cm^−2^ at 50 °C, 122% higher than the pristine membrane. 

Recently, DABCO quaternized poly(arylene ether) (QPAE) was filled with ammonium functionalized GO (GO-(APTS-c-PTMA)) using (3-aminopropyl)triethoxysilane (APTS) and (3-bromopropyl)trimethyl ammonium bromide (PTMA) as chemical cross linkers [83]. The hybrid with 0.7 wt% of filler showed an OH^−^ conductivity of 114 mS cm^−1^ at 90 °C and the remaining ionic conductivity, after soaking in 2 M KOH at 80 °C, was 75% of the initial value. The H_2_/O_2_ FC performance at 70 °C and 100% RH showed a maximum power density of 136 mW cm^−2^ and a current density of 317 mA cm^−2^.

#### 3.3.6. Other Aliphatic Polymers

Some aliphatic polymeric matrices not containing ether bonds were explored, with the aim of decreasing degradation under alkaline conditions.

**Figure 9:**
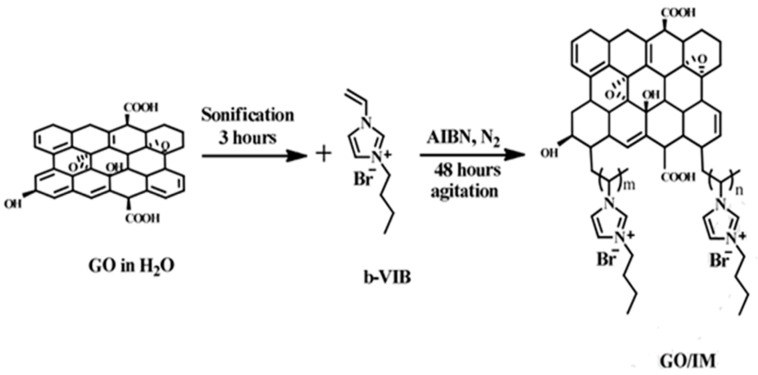
Modification process of GO via free radical polymerization-grafting with a b-VIB group. Reproduced with permission from Ref. [87].

Liu et al. synthesized a perfluorinated anion-conducting polymer (I-PFSO_2_NH_2_-Cl) in 2017 with ionic liquid functionalized graphene nanoribbons (IGNRs). The composite with 1.0 wt% of filler attained a conductivity of 121 mS cm^−1^ at 80 °C in liquid water. The single cell performance with IGNRs/Pt electrocatalysts showed a maximum power density of 197 mW cm^−2^ and a current density of 372 mA cm^−2^ [84]. Cross-linked quaternized poly(styrene-b-isobutylene-b-styrene) (QSIBS) was the host matrix for organo-modified graphene oxide (GOAN) quaternized with TMHDA [85] and for covalently linked graphene [86]. GO modified with butylvinylimidazolium (GO/IM) was incorporated inside a complex matrix composed by para-methyl styrene/butylvinylimidazolium (PMS/b-VIB) and poly(4,4′-diphenylether-5,5′-bibenzimidazole) (DPEBI) [87]. The synthesis of GO/IM is reported in Figure 9. The nanohybrid membranes attained an OH^−^ conductivity of 102 mS cm^−1^ at 100 °C. The activation energy was between 16 and 19 kJ mol^−1^ corresponding to a Grotthuss-type mechanism; the lower value was observed for 2 and 3 wt% filler content. 

A GO multilayer paper was also employed as a matrix. The membranes were treated with KOH and used in FC [88].

### 3.4. Carbon and Boron Nitride (BN)

Recently, some examples were reported in the literature on the use of nitrides, characterized by extreme hardness, to improve the mechanical properties and decrease the crossover of membranes.

Graphitic carbon nitride (g-C_3_N_4_) nanosheets were used as filler in modified quaternary aminated poly(arylene ether sulfone) (QPAES) [89]. The Young’s moduli were in the range of 1150–1670 MPa and increased with respect to bare QPAES (1110 MPa). AEMFC tests at 60 °C, for 0.6 wt% filler, showed the highest power density with 68 mW cm^−2^. g-C_3_N_4_ nanosheets were also inserted into PAEK functionalized by Menshutkin reaction with TMA (QPAEK) [90]. The Young’s modulus and the TS increased as expected; the maximum power density in a single H_2_/O_2_ FC was reached for the QPAEK-CN-0.5 membrane with 49 mW cm^−2^ at 80 °C. PPO quaternized with N-methyl morpholine was used by Rathod et al. in 2020 to prepare composite membranes with a different amount (1–5 wt%) of functionalized boron nitride (BN) [91]. Membranes with 5 wt% exhibited the best performances; the ionic conductivity was acceptable (6.3 mS cm^−1^ at 30 °C) and the MeOH permeability decreased (5.15 × 10^−8^ cm^2^ s^−1^) indicating that BN acted as a barrier for methanol limiting the cross-over. 

Table 3 summarizes information on the various composites, including the type of polymer and other 2D filler, remarks and references.

We have seen in this section various 2D nanofillers, such as LDH, MXene, graphene and graphene oxide, etc., commonly used for the fabrication of hybrid materials especially with commercial and low cost polymers such as PPO and PSU. The purpose was to maintain good conductivity, also by functionalizing the inorganic fillers with ionic conducting groups, while trying to enhance the mechanical properties and reduce the permeability of gases in the FC, thanks to the 2D geometry of these materials.

## 4. 3D Materials

### 4.1. Silica and Silicates

Silicon dioxide and silicates are the most abundant classes of minerals in nature, and their cost is practically negligible. Phyllosilicates, such as montmorillonite and palygorskite, are hydrated silicates of aluminium and/or magnesium with the ability to exchange ions. Notable successes were obtained by using Si and its derivatives as fillers in proton exchange membranes [92,93]; later their use was extended to AEM. In this review, we will limit the description of the progress achieved to silica and derivatives not obtained via the sol–gel process. Sol–gel silica composites were described in a recent review by Sgreccia et al. [94]. 

#### 4.1.1. Poly(vinylidene fluoride) (PVDF) and Poly(vinyl alcohol) (PVA)

One of the first examples was proposed by Zuo et al. who prepared a composite with TMA-PVDF and SiO_2_ [95]. The membrane with 2 wt% of SiO_2_ showed the best specific conductivity (3 mS cm^−1^). More recently silica-coated PVDF (SiO_2_@PVDF) electrospun nanofibers were quaternized and inserted in QCS [96]. Tests in alkaline DMFC, performed at various MeOH concentrations, showed a maximum power density at 80 °C up to 99 mW cm^−2^ (2 M MeOH), presenting only 4% of performance loss after 100 h in chronoamperometry test.

PVA was often used as matrix for silica hybrids [97,98]. A DMAFC maximum power density (97 mW cm^−2^, 80 °C with 2 M MeOH + 6 M KOH) was achieved with quaternized PVA and 5 wt% of nanosized (14 nm) fumed silica (FS) due to its higher free volume and hydroxide ion transfer phenomenon [99]. A different approach was proposed by Lu et al. where cellulose nanocrystal (CNC)-based composite films were mixed with 40% of hydrophobic binder (PVA : silica gel = 1:2) [100]. Due to the hydrophilicity and dimensional stability of CNCs, membranes exhibited high water uptake (~80%) but low water swelling (~5%).

For PEM electrochemical reactors, a blend of PVA and CS was doped with organic ionomers (4VP, methyl chloride quaternary salt resin), commercial ionomer filler (AS4, structure not disclosed by the industry), inorganic titanosilicate (AM-4, containing Na^+^ ions) and layered stannosilicate (UZAR-S3, Na_7_Sn_2_Si_9_O_25_) [101]. The best performances were obtained for UZAR-S3/CS:PVA and 4VP/CS:PVA composites. 

#### 4.1.2. Aromatic Polymers

**Figure 10:**
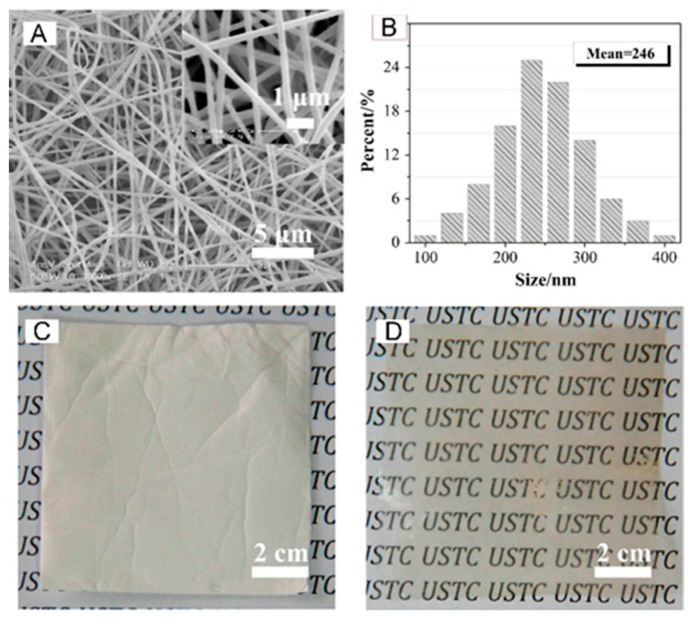
SEM images (**A**) and fiber diameter distribution (**B**) of QPPO–SiO_2_; optical photographs of nanofiber mats (**C**) and membrane (**D**) after hot-press. Reproduced with permission from Ref. [102].

Aromatic polymers were also a preferred choice in the building of silica-containing membranes for fuel cells, diffusion dialysis (DD), and redox flow batteries. Pan et al. in 2015 improved the efficiency of acid recovery by DD with the fabrication of a quaternized PPO/SiO_2_ hybrid material (QPPO–SiO_2_) obtained by electrospinning and post-treatment (solvent fumigation and hot-press) [102]. The main fiber diameters were between 200–300 nm with an average of ~250 nm as shown in Figure 10.

In a simulated polishing waste solution containing 1 M HCl and 0.225 M FeCl_2_, the membrane exhibited both a higher acid permeability (UH) with 0.053 m h^−1^ and selectivity (S, 68.05) in comparison with the direct casting QPPO–SiO_2_ hybrid membrane. In addition, compared with a commercial DF-120 membrane the hot-pressed electrospun QPPO–SiO_2_ membrane showed more than seven times higher UH and approximately three times higher S, underling its advantage in DD application [102]. Quaternized polyethersulfone (TMA-PES) composite membranes were fabricated with the incorporation of three functionalized SiO_2_ nanoparticles containing propylamine (ASi-I), trimethylpropylamine (ASi-II) and melamine-based dendrimer amine groups (ASi-III) [103].

Recently Chen et al. prepared composites with a “hamburger structure” using quaternized PPO with a triple-cation precursor (TA-PPO) as matrix and 1,2-dimethylimidazolium-silica (Im-SiO_2_) as dopant. Im-SiO_2_ was placed on the surface of TA-PPO membrane to protect the matrix from the attack of OH^−^ and radicals [104]. The composite membrane showed a low swelling ratio (8.2%), and a high OH^−^ conductivity (105 mS cm^−1^ at 80 °C).

Quaternized mesoporous silica nanoparticles (QMSNs) were mixed with PSU quaternized with TEA [105] and TMA [106]. The homogeneous dispersion of QMSN in the QPSU matrix, highlighted by the SEM images, improved the mechanical properties: the Young’s modulus increased from 1990 for pure QPSU to 2250 MPa for 20 wt% of QMSNs while the composite with 15 wt% of filler showed the highest CO_3_^2−^ conductivity, reaching 20 mS cm^−1^ at 80 °C [106]. Afterwards mesoporous silica (SBA-15) was functionalized with imidazolium-based ionic liquid and inserted in QPSU. The presence of covalent bonds between IL and SBA-15 was verified by solid-state NMR [107]. The QPSU/3%IL-SBA-15 showed the best fuel cell performance with an OCV of 0.87 V and a maximum power density of 278 mW cm^−2^. 

TMA-PSU was a host membrane for two functionalized montmorillonites, containing cetyl trimethyl ammonium chloride (MMT-1) and (3-aminopropyl) triethoxysilane (MMT-2) [108]. The membrane containing 5 wt% of MMT-1 showed the highest conductivity with 47 mS cm^−1^ at 95 °C. The alkaline stability was evaluated in 2 M NaOH for 120 h at 60 °C and the residual ionic conductivity was around 80% [108]. TMA-PSU (QPSU) was also used as matrix for natural hydrophilic “nanofiber-like” palygorskite (Pal) particles [109]. SAXS showed a good hydrophilic–hydrophobic microphase separation that led to a high OH^−^ conductivity and QPSU/Pal-0.5 reached 93 mS cm^−1^ at 80 °C. The TS was between 24 and 45 MPa, the Young’s modulus 1177–1848 MPa and the elongation at break 3–12%. 

**Figure 11:**
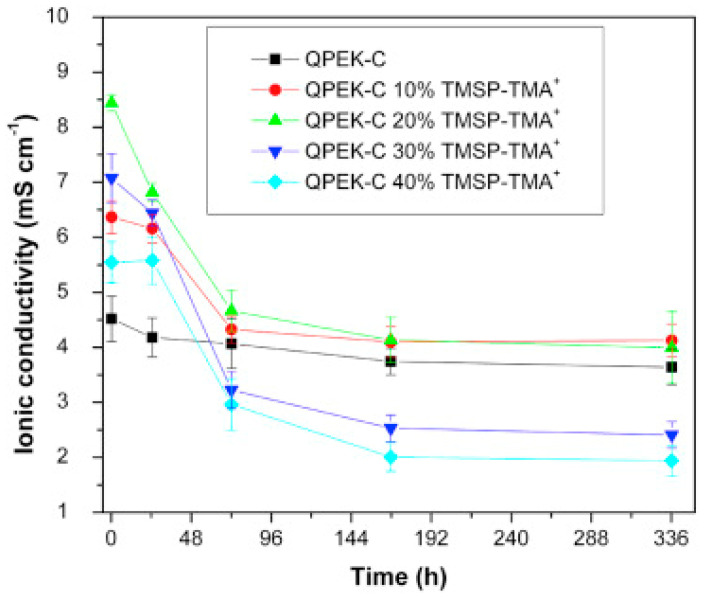
Effect of exposure to (1.5 M VO_2_^+^ + 3 M H_2_SO_4_) at 30 °C on sulfate ion conductivity of QPEK-C and QPEK-C/10–40 wt% TMSP-TMA^+^ composite membranes. Reproduced with permission from Ref. [110].

Another aromatic polymer, quaternized cardo-poly(ether ketone) (QPEK-C) were doped with a quaternized ORMOSIL (TMSP-TMA^+^Cl^−^) [110]. The composites were used in all vanadium redox flow batteries; Figure 11 shows the sulfate ion conductivity with different percentages of TMSP-TMA^+^ as a function of time. The best results were obtained with 10–20 wt% of TMSP-TMA^+^.

In VRFB, the CE at 100 mA cm^−2^ was 99% for both, QPEK-C and QPEK-C/20 wt% TMSP-TMA^+^, compared to 95% for Nafion^®^ 212. The battery capacity was 10% lower over 30 charge/discharge cycles (~60 h) while for Nafion^®^ 212 in similar conditions a loss of 30% was observed [110]. 

Table 4 summarizes information on the various composites, including the type of polymer and 3D filler, remarks and references.

### 4.2. Metal Oxides and Derivatives

One of the major uses of metal oxides as filler in AEM is in improving the mechanical properties of soft polymers such as PVA. Various oxides are used, including Al_2_O_3_, a well known hard material characterized by poor thermal and electrical conductivity, TiO_2_, non-expensive and non-toxic with high chemical stability, and ZrO_2_, a polymorphous crystalline oxide with high ionic conductivity when doped with acceptor cations and low electronic conductivity. The chosen matrices were mainly PVA and aromatic polymers.

#### 4.2.1. Aluminum Oxides

**Figure 12:**
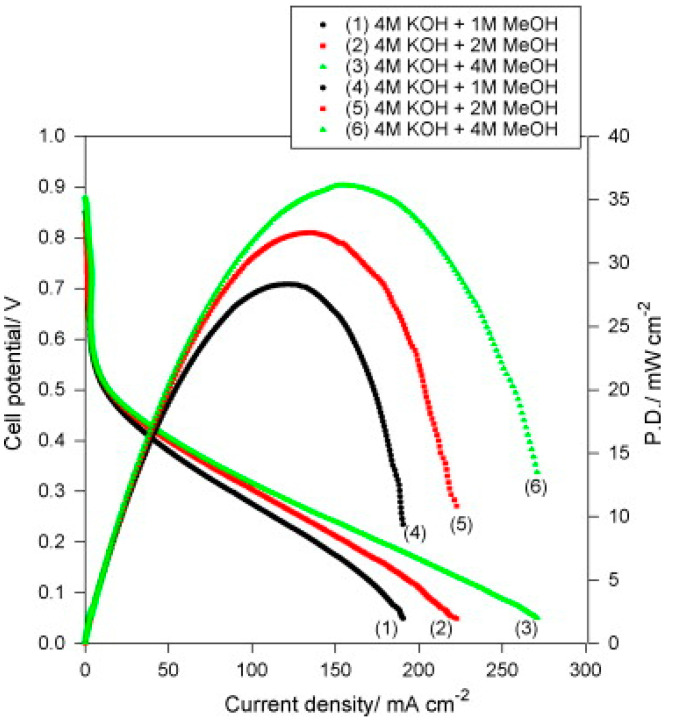
DMFC curves of QPVA/10 wt% Al_2_O_3_ composite membrane with various fuels (4 M KOH + x M CH_3_OH) at 25 °C and in ambient air. Reproduced with permission from Ref. [111].

PVA quaternized with glycidytrimethyl ammonium chloride and 10 wt% Al_2_O_3_ (QPVA/Al_2_O_3_) displayed in DMAFC a maximum power densities of 36 mW cm^−2^ with 4 M KOH + 4 M CH_3_OH, as shown in Figure 12 [111]. Although SEM images indicated some chunks and aggregates randomly distributed on the surface, the storage modulus slightly increased with respect to pure PVA (172 vs. 151 MPa at 100 °C).

Composite membranes with smooth and dense morphology were formed by quaternary trimethylammonium PSU with different loadings of Al_2_O_3_ powder (1–4 wt%) [112]. The presence of filler increased the swelling degree, the water uptake, and the ionic conductivity.

#### 4.2.2. Zirconium Oxides

The morphology of composite membranes of TMA-PSU and zirconia was investigated by TEM, revealing a homogenous distribution when the particles size were around 10 nm [113]. The membrane with 10 wt% of ZrO_2_ showed the best performance, reaching a maximum conductivity of 15 mS cm^−1^. The maximum power density presented a value of 250 mW cm^−2^ with an OCV of 0.91 V at 60 °C [113]. Based on Im-PSU, recently, Rambabu et al. prepared composites with different percentages of zirconia (2.5, 5, 7.5 and 10 wt%), through a phase inversion method [114]. The conductivity attained was 80 mS cm^−1^ at 50 °C, 47% higher than pure Im-PSU. The TS showed values of 47 and 43 MPa before and after the alkaline treatment, while pure Im-PSU presented 31 and 25 MPa, respectively. The FC performance, using Pt/C catalysts, showed with 10 wt% of ZrO2 an OCP of 1.04 V, a maximum power density of 270 mW cm^−2^ and a current density of 640 mA cm^−2^. 

Another quaternized aromatic polymer (QPAES) was used to prepare composite membranes with nanozirconia [115,116]. The introduction of the second phase enhanced the OH^−^ conductivity of cross-linked multiblock copoly(arylene ether sulfone) (XL TMA-coPAES), with 7.5 wt% of ZrO_2_ the composites showing the best conductivity (55 mS cm^−1^ at 80 °C) and the best alkaline stability, retaining 94% of conductivity after immersion in 1 M NaOH solution at 60 °C for 340 h. The highest Young’s modulus was reached for 10 wt% of ZrO_2_ with 492 MPa, around two times higher than virgin polymer. Fluoropolymer-based membranes were obtained from a free-radical desulfurization coupling reaction on Nafion NR-50 followed by grafting and filling with ZrO(ClO_4_)_2_ [117]. The chemical stability was evaluated in a reactor with a 7 M KOH solution of EtOH/H_2_O 15%/85% at 120 °C for 2200 h. The IEC after the stability test was practically unchanged. The high stability was ascribed to the fluorinated polymeric structure and to covalent C–C bonds that link the ZrO(ClO_4_)_2_ nanoclusters and polymer side chains [117]. 

#### 4.2.3. Titanium Dioxide and Titanates

Many aromatic polymers were used as host matrices: PSU and quaternized polystyrene-block-poly(ethylene-ran-butylene)-block-polystyrene (PSEBS) [118], TMA-PSU [119], vinylbenzyl chloride-divinylbenzene copolymers (AEH) [120], PSU and TMA-PPO [121]. 

The effect of the doping with hydrophilic (tri(hydroxymethyl)propane, TMP) and hydrophobic (polymethyl-hydrosiloxane, PMHS) TiO_2_ nanoparticles was studied using DABCO-PSU as the matrix [122]. The DMA and DSC studies showed a Tg around 250 °C, due to a partial crystallization of the polymer, with a significant decrease in the contact angle (67°) for composites with hydrophilic character. The ionic conductivity was higher for the hydrophobic filler (PMHS-TiO_2_) related to a more homogeneous dispersion, as observed by AFM images. 

Some examples of composites concern the use of ionic liquids and nanoparticles with the aim of simultaneously increasing conductivity and mechanical properties. 1-Methy-3-methylimidazolium IL and nano-TiO_2_ were mixed to TEA-PPO [123]. The conductivity increased with the amount of IL and TiO_2_ and the best mechanical properties were reached for 15 wt% IL and 1 wt% of TiO_2_ with Young’s modulus of 921 MPa and 6% of elongation at break. The degradation rates of the ion conductivity measured after treatment in 4 M NaOH solution at RT for 280 h, showed for 1 wt% of TiO_2_ a reduction of around 30% instead of 80% without the nanofiller, confirming the role of TiO_2_ in stabilizing the IL in the membranes [123]. The same authors inserted methyl, ethyl, hydroxyethyl IL and TiO2 into a TEA–PPO matrix [124]. The stability tests, in 4 M NaOH at RT for 288 h, revealed for the ethyl derivative a 92% retention of conductivity while the pristine membrane retained 78%. 

PVA based-membranes were used in microbial fuel cells (MFCs) [125]. The comparison between TiO_2_–PVA quaternized with trimethylammonium chloride (QAPVA), commercial Nafion 117, and LeHoAM-III (Hangzhou Lvhe Environmental Technology Co., Ltd., China) showed a maximum power density at 35 °C for TiO2-QAPVA (125 mW cm^−2^), two and three times higher than Nafion 117 and LeHOAM-III, respectively. The TiO_2_-QAPVA superior performances were attributed to a good oxygen resistance of the membrane. 

PVA membranes, filled with spherical calcium titanate nanoparticles (CaTiO_3_) with orthorhombic perovskite structure, were proposed for redox flow batteries [126]. The distribution was uniform and homogenous when the nano-CaTiO_3_ filler was less than 20 wt%.

### 4.3. Other Inorganics 

Homogeneous composite membranes with PTFE and Sn_0.92_Sb_0.08_P_2_O_7_ exhibited conductivities from ~10 to 100 mS cm^−1^ between 75 and 200 °C [127]. In the fuel cell tests, the maximum power density reached 94 mW cm^−2^ at 100 °C. 

**Figure 13:**
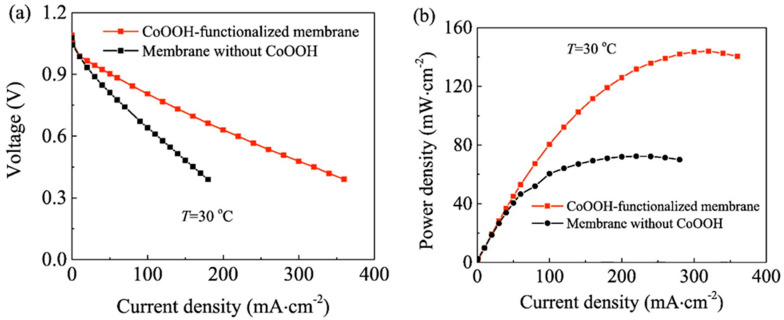
DBFCs performances for CoOOH^−^ functionalized and pristine membranes. V (**a**) and power density (**b**) as a function of discharge current density at 30 °C. Reproduced with permission from Ref. [128].

In Direct Borohydride Fuel Cells (DBFCs), PVA membranes with CoOOH^−^ functionalization showed a better performance than pristine membranes [128]. The maximum power densities obtained in the test cells at 30 °C with and without CoSO_4_ were 144 and 72 mW cm^− 2^, respectively (Figure 13). 

PVA was employed to prepare composites based on TMA-PVA/chitosan/molybdenum-disulfide (QPVA/CS/MoS_2_) with different amounts of MoS_2_ nanosheets [129]. The membranes with 1.0 wt% MoS_2_ showed a TS of 33 MPa. The MeOH permeability of pure QPVA/CS was 1.0 × 10^−7^ cm^2^ s^−1^ and decreased to 0.2 × 10^−7^ cm^2^ s^−1^ with the addition of 1.0 wt% of MoS_2_. 

Zinc oxide nanoparticles (ZnO) were used as fillers in blended TMA-PPO and PSU [130]. The maximum power density at RT was 69 mW cm^−2^ (current density 220 mA cm^−2^), three times high than pure QPPO at the same conditions. 

AEM with flame-resistance properties were prepared with PBI and 1-butyl-3-methyl imidazolium phosphotungstate (PWA-IL) [131]. SEM evidenced an irregular crystal structure of PWA-IL hybrids. After modification, the PBI/(PWA-IL) showed a lower TS (65 MPa) than pristine PBI (80 MPa), while the elongation at break (PWA-IL 1:4) increased from 6% to 13%. The anionic conductivity at 80 °C of PBI/(PWA-IL 1:5) containing 20 wt% of PWA-IL, was 76 mS cm^−1^, higher than pristine PBI (36 mS cm^−1^). 

### 4.4. Metal Organic Frameworks (MOFs)

Crystalline metal–organic frameworks (MOFs) are formed by metal ions coordinated with rigid organic ligands to form high porosity structures and are therefore characterized by very large internal surface areas. With tunable functionality and well-defined channels, MOFs have inspired a new class of ion-conductive compounds. In contrast to the extensive studies on proton-conductive MOFs [132] and related membranes, rare reports focus on MOFs in the preparation of AEM.

**Figure 14:**
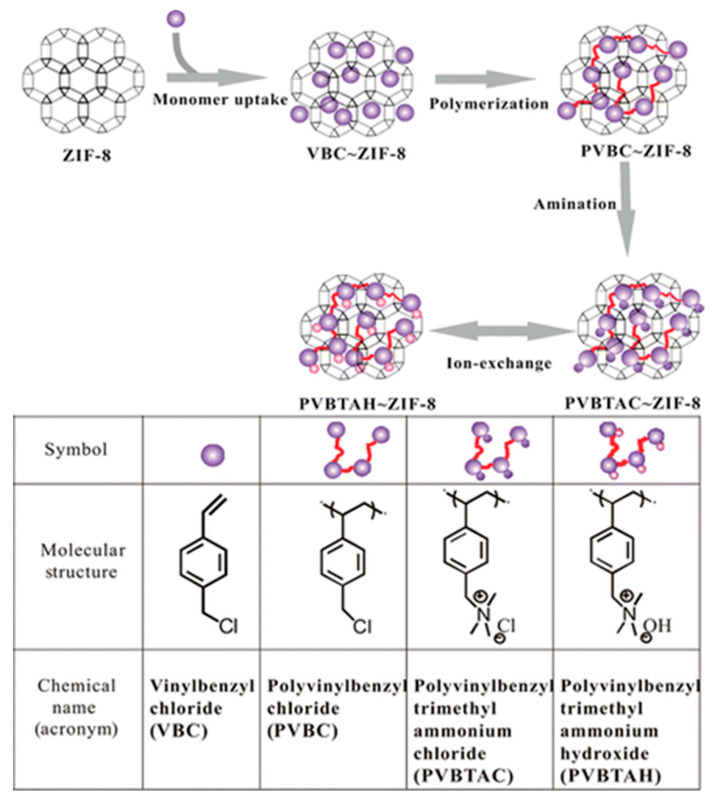
Synthesis of ion-exchange polymer (PVBTAH) inside the porous network of ZIF-8. Reprinted with permission from Ref. [133]. Copyright (2014) American Chemical Society.

Vinyl benzyl chloride (VBC) monomers were impregnated into nanopores of zeolitic imidazolate framework (ZIF-8) and then aminated to obtain poly vinyl benzyl trimethylammonium chloride (PVBTAC) as described in Figure 14 [133]. The Brunauer–Emmett–Teller (BET) surface analysis showed, after incorporation of the polymer, a pore volume of ZIF-8 from 0.49 to 0.26 mL g^−1^. The absence of a cross-linking agent in the polymerization ensured the formation of linear chains in the structure. 

ZIF-8 was also added in a different amount to PVA [134]. To enhance the electrochemical properties at low humidity, choline hydroxide ionic liquids were used in PVA-ZIF-8 system [135]. The conductivities of IL@ZIF-8/IL/PVA composites were tested at 33% of RH and improved from 0.11 mS cm^−1^ at 25 °C to about 1 mS cm^−1^ at 60 °C for 20 wt% of IL@ZIF-8. 

PVA-ZIF-8 composite membranes were also used for DMAFCs [136]. The alkaline stability test for PVA-40.5% ZIF-8 after immersion in 6 M KOH for 24 h and 168 h showed a decrease in conductivity by 14%, for pure PVA 31%. For the same composition, a maximum power density of 173 mW cm^−2^ was achieved in DMAFC at 60 °C. 

A sandwiched AEM formed by porous bromomethylated-PPO with entrapped cationic MOFs coated with PVA on the two sides was prepared for DMAFCs [137]. PVA coating limited the MeOH crossover and entrapped cationic MOFs worked as OH^−^ conductive channels. The OH^−^ conductivity was 145 mS cm^−1^ at 80 °C and the MeOH permeability was 3.68 × 10^−7^ cm^2^ s^−1^ [137]. 

The incorporation of chloromethylated MIL-101(Cr) into chloromethylated PEEK, followed by quaternization with imidazolium, gave Im-PEEK/ImMIL-101(Cr) membranes [138]. ImMIL-101(Cr) was uniformly distributed into the Im-PEEK matrix up to 1.0 wt%; above this value a slight aggregation of the filler was observed. The TS of the composite membranes reached the highest value of 35 MPa with the incorporation of 10 wt% of ImMIL-101(Cr), 47.5% higher than pure Im-PEEK, the presence of filler reducing the elongation at break and increased the rigidity of membranes. 

### 4.5. Carbon Dots (CDs)

Although the CD particle is classified with zero dimensions, we will deal with it in this section for simplicity.

**Figure 15:**
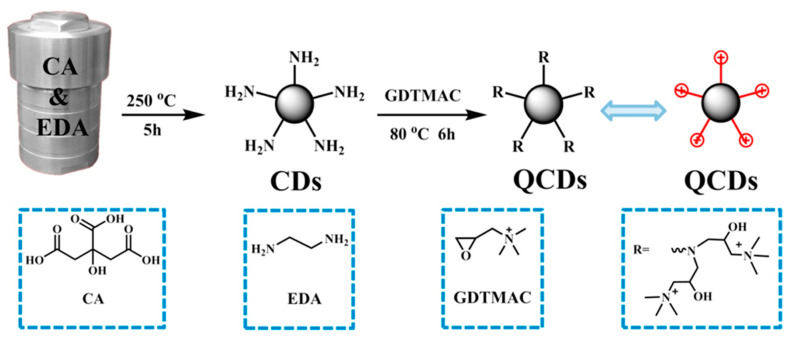
Synthesis of CDs and QCDs. Reproduced with permission from Ref. [139].

Yuan et al. in 2019 prepared nanocomposites formed by an Im-PSU matrix and quaternized carbon dots (QCDs) derived from citric acid and ethylenediamine as shown in Figure 15 [139]. 

The Young’s modulus rose to 1600 MPa; the TS 70 MPa and the elongation at break rose to 15.5 %. For Im-PSU-1.0%-QCDs, the stability tests showed 61% of OH^−^ conductivity retention, (original value 109.3 mS cm^−1^), after 500 h in 1 M NaOH at 60 °C. 

Composite AEMs based on chloromethylated PSU were developed for the DD process in the recovery of acid from the waste stream in 2020 [140]. The composites were prepared with different wt% (0.1, 0.5, and 1.0) of graphene quantum dots (GQDs) synthesized by chemical oxidation of MWCNTs, followed by quaternization with TMA (called SCE-0.1, SCE-0.5, and SCE-1.0). The conductivity for SCE-0.1 attained was 15 mS cm^−1^. TS and elongation at break increased, respectively, by 37% and 28% for a composite with 1.0 wt% GQDs with respect to pristine SCE. Acid recovery with 3 M HCl solutions was 30% for pristine membranes, while for SCE-1.0 it increased to 44%. 

Table 5 summarizes information on the various composites, including the type of polymer and other 3D filler, remarks and references.

We have seen in this section different 3D nanofillers such as silica, ZrO_2_, MOF and CD commonly used for fabrication of hybrid materials especially with commercial, low-cost polymers such as PPO, PSU and very attractive PVA. The greatest efforts have focused on silica and functionalized derivatives and oxides, especially zirconia. The purpose was to increase conductivity, slightly improve IEC, enhance mechanical properties especially Young’s modulus and increase the power density in FC tests. Generally, the stability in an alkaline environment improves, but not significantly except for fluoropolymer-based anion-conducting membranes containing ZrO(ClO_4_)_2_ where the stability seems to exceed 2000 h.

## 5. Conclusions

This review on composite anion exchange membranes with inorganic fillers allows some general conclusions. We divided the results according to the dimensionality of the fillers. Some interesting synthetic efforts versus the innovative functionalization of nanoparticles can be recognized. Generally, an enhancement of mechanical properties (TS, Young’s modulus) is observed in composites. The ionic conductivity is often reduced with some possible mitigation by an intrinsic ion conducting fillers or little improvement with functionalized fillers.

Frequently an enhancement of FC performances is reported, but not much improvement on membrane stability in alkaline conditions. 

For 1D fillers, the major work was perfromed on carbon nanotubes with various functionalization and some papers on titanate nanotubes. One-dimensional tubular nanofillers can provide ion conducting groups, construct ion conducting pathways along the interphase region and improve the anti-swelling property of the nanohybrid membrane. The carbon nanotubes are also characterized by extreme strength and flexibility; the increase in mechanical properties is evident but the increase in FC performance is modest. The use of imidazole as functionalizing molecule contributes to attainment of a high conductivity; a composite with imidazole PEEK and imidazole CNT reached a hydroxide conductivity of 135 mS cm^−1^ at 70 °C.

For 2D fillers, by far the largest amount of results was reported on graphene oxides; significant work was also performed on inexpensive layered double hydroxides which are intrinsically anionic conductors. A perfluorinated anion-conducting polymer with ionic-liquid-functionalized graphene nanoribbons attained a conductivity of 121 mS cm^−1^ at 80 °C in water. Sandwiched-porous PBI and reduced graphene oxide attained a maximum power density at 90 °C of 544 mW cm^−2^. The membrane GO/cellulose/PPO displayed a conductivity of ~114 mS cm^−1^ at 25 °C and ~215 mS cm^−1^ at 80 °C. PVA and exfoliated LDH(NO_3_^−^) membrane exhibited a conductivity of 172.1 mS cm^−1^ at 80 °C. A porous-sandwich structure based on triple-cation side chain PPO and quaternary-ammonium-modified LDH exhibited a maximum power density of 267 mW cm^−2^ at 60 °C.

For 3D fillers, most works were reported on functionalized silica and zirconia nanoparticles. The ionic conductivity generally did not improve much, remaining at acceptable values around 10 mS cm^−1^ at 60 °C for most composites with silica. A maximum power density of 298 mW cm^−2^ at 60 °C was attained for a QPSU with triethylamine functionalized mesoporous silica. The stability in an alkaline environment was improved, but not significantly except for a fluoropolymer-based AEM containing ZrO(ClO_4_)_2_ where the measured stability seems to exceed 2000 h. As for the MOFs, we can see a good conductivity for MIL 101 with 145 mS cm^−1^ at 80 °C.

Still open challenges that must be overcome for a large-scale application of AEMFCs as alternative to PEMFCs are: (i) the creation of alkaline stable ionomers, (ii) the creation of efficient and stable non-PGM catalysts for HOR/ORR necessary to make a low-cost final device. Other points must be optimized including the components of the MEA and their assembly, support for the catalyst, etc. As far as composites are concerned, the improvement from a mechanical point of view is evident, but they still suffer from low alkaline stability at high temperatures. Other points of discussion are certainly: (i) clarifying the interaction at the interface between the composite membrane and the catalyst, (ii) how to improve water management, (iii) how to avoid carbonation and (iv) the use of easily reproducible test conditions for a true comparison of membrane performances. Concerning the composite membranes reported in the review, few cases are highlighted with relatively new materials such as MOFs, CDs and MXenes; we expect in the future an increase in the use of these materials by optimizing their properties as needed and making them more stable, e.g., with the formation of covalent bonds between organic and inorganic phases. Another point to explore is replacing the commercial AEM that suffers from a significant degradation both at the level of the cationic group and of the backbone, with more stable polymers such as polynorbornene, polystyrene, and ionic groups, such as n-dimethyl piperidinium-type. Even if these materials are more expensive, this disadvantage can be mitigated by a future wide commercialization of AEM capable of operating with high current densities at temperatures of 80–95 °C.

## Figures and Tables

**Table 1:** Type of polymer and 1D filler in composite AEM with TNT and CNT.

Polymer	1D Filler	Remark	Ref
TEA-PSU	triethylammonium TNT	maximum power density 285 mW cm^−2^ at 60 °C	[20]
TEA-PSU	1-methyl-3-(3-trimethoxysilylpropyl) Im-chloride TNT	302 mW cm^−2^ at 60 °C (5 wt%)	[21]
TMA-PAEK	trimethylammonium (3-chloropropyl)-trimethoxysilane TNT	conductivity 52.5 mS cm^−1^ at 80 °C	[22]
CS	QHNT	TS 52 MPa, Young’s modulus 1100 MPa (5 wt%)	[23]
PVA	MWCNT	methanol permeability 3.57 × 10^−7^ cm^2^ s^−1^	[24]
PVA	grafted MWCNT	KOH 8 M solution uptake 108 %	[25]
PVA	FeCNT	maximum power density 88 mW cm^−2^ at 60 °C (DMAFC)	[26]
PVA	grafted MWCNT	maximum power density 65 mW cm^−2^ at 60 °C (DEAFC)	[27]
PVA/poly(diallyldimethylammonium chloride)	MWCNTs-OH	TS 40 MPa (3 wt%)	[28]
CS	MWCNTs-OH	conductivity 6 mS cm^−1^ at RT	[29]
glycidyltrimethylammonium chloride-CS (GTA, AR)	CNT	TS 32 MPa	[30]
2,3-epoxypropyl trimethyl ammonium chloride-CS	QSiO_2_-CNT	maximum power density 81 mW cm^−2^ at 60 °C (DMAFC)	[31]
Q-trimethylamine polystyrene-block-poly(ethylene-ran-butylene)-block-polystyrene	TMA-MWCNT	maximum power density 187 mW cm^−2^ at 60 °C (DMAFC)	[32]
Im-PPO	PIL(BF_4_) MWCNT	conductivity 56 mS cm^−1^ at 75 °C	[33]
Im-PEEK	Im-CNT	conductivity 135 mS cm^−1^ at 70 °C, 100% RH	[34]
Im-PEEK	poly(vinyl imidazole)-CNT	maximum power density129 mW cm^−2^ at 60 °C (AEMFC)	[35]
Im-PSU	Im-MWCNTs	TS 24 MPa (0.2 wt%)	[36]

**Table 2:** Type of polymer and 2D filler in composite AEM with LDH or MXene.

Polymer	2D Filler	Remarks	Ref
(TMA, DABCO)-PSU	MgAl-LDH (Cl^−^)	Young’s modulus 620 MPa (100% RH)	[37]
XL glutaraldehyde-PVA	MgAl-LDH (CO_3_^2−^)	EtOH permeability 1.8 × 10^−7^ cm^2^ s^−1^	[38]
2,3-epoxypropyltrimethylammonium chloride-PVA	MgAl-LDH(CO_3_^2−^, NO_3_^−^)	NO_3_^−^ conductivity 156 mS cm^−1^ at 80 °C, TS 48 MPa	[39]
glycidyltrimethylammonium chloride-CS/PVA	MgAl-LDH (NO_3_^−^)	TS 24 MPa	[40]
(2,3-epoxypropyl trimethyl ammonium chloride)-CS/PVA	CNT coated with MgAl-LDH (CO_3_^2−^)	maximum power density 107 mW cm^−2^ (DMAFC)	[42]
glycidyl trimethyl ammonium chloride-CS/PVA	MgAl-LDH (CO_3_^2−^) wrapped on quaternized SiO_2_ nanospheres	CO_3_^2−^ conductivity 11 mS cm^−1^ at 80 °C	[41]
TMA-PSU	MgAl-LDH (NO_3_^−^)	TS 21 MPa	[43]
DABCO-PSU	ZnAl-LDH (BmimSO_4_)	conductivity 16 mS cm^−1^ at 25 °C	[44]
1-methylimidazole PPO	MgAl-LDH (CO_3_^2−^)	tensile strength 29.5 MPa	[47]
1-(N′,N′-dimethylamino)-6,11-(N, N,N-trimethylammonium) undecane-PPO	MgAl-LDH with N,N,N-trimethylpropyltriethoxysilane ammonium chloride	conductivity 122 mS cm^−1^ at 80 °C, maximum power density 267 mW cm^−2^ at 60 °C	[46]
1-(N′,N′-dimethylamino)-6-(N,N′-dimethylammonium)-11-(N,N′,N″-trimethyl ammonium)undecane-PPO (TC-PPO)	MgAl-LDH, 3-hydroxy-6-azaspiro [5.5] undecane, N,N,N-trimethyl-3-(triethoxysilyl)propan-1-aminium bromide (ASU-LDH)	IEC values 3.11–3.90 meq g^−1^	[45]
TC-PPO	MgAl-LDH (ASU-LDH) electric-field-aligned	conductivity 110 mS cm^−1^ at 80 °C	[48]
DABCO-PPO	MgAl-LDH	Young’s modulus 320 ± 60 MPa (100% RH)	[49]
electrospun PVDF	Mg_4_Al_2_(OH)_12_CO_3_·3H_2_O	conductivity 87 mS cm^–1^ at 70 °C, 100% RH	[50]
triethylene diamine-PSU	LiF-Ti_3_C_2_T_x_; NH_4_HF_2_-Ti_3_C_2_T_x_	maximum power density 101 mW cm^−2^ at 60 °C	[52]
CS	Im brush-functionalized MXene	TS 41.0 MPa (7.5 wt%)	[53]

**Table 3:** Type of polymer and 2D filler in composite AEM with graphene, GO, carbon nitride or boron nitride.

Polymer	2D Filler	Remarks	Ref
PVA	exfoliated graphene	maximum power density 46 mW cm^−2^ at 60 °C	[54]
PVA/CS	graphene, sulfonated graphene	conductivity 47 mS cm^−1^ at 25 °C	[55]
QPEI/PVA	silica functionalized GO	conductivity 72 mS cm^−1^ at 30 °C	[56]
XL glycidyl-trimethylammonium chloride-PVA	GO	EtOH permeability 3.65 × 10^−7^ cm^2^ s^−1^ at 60 °C	[57]
glycidytrimethyl ammonium chloride PVA	aligned GO-Fe_3_O_4_	maximum power density 172 mW cm^−2^ at 60 °C (0.1 wt%)	[58]
glycidytrimethyl ammonium-PVA (QPVA)	GO-Fe_3_O_4_	conductivity 47–55 mS cm^−1^ at 30–60 °C (0.1 wt%)	[59]
TMA-PSU	QGs	TS 205 MPa (0.25 wt%)	[60]
TMA-QPSU	dopamine modified GO (DGO)	TS 13 MPa, elongation at break 33% (1 wt%)	[61]
TMA-QPSU	XL-GO	swelling ratio 3.9%, water uptake 19% at 60 °C (2 wt%)	[62]
diethanolamine-modified PSU (HPSU)	guanidinium-GO	swelling of 9% at 60 °C	[63]
3-azidopropyl-N,N-dimethylamine (Ap-DMA) and TMA (AMPSU) QPSU	(azide XL) rGO	Young’s modulus 2890 MPa, RH 100% (0.1 wt%)	[64]
TMA-PPO/PSU	GO	IEC 3.21 meq g^−1^ (2 wt%)	[65]
Im-functionalized bisphenol PSU	Im-GO	maximum power density 79 mW cm^−2^ at 60 °C (0.2 wt%)	[66]
TMA-PSU	QPbGs (polymer brush funzionalized graphenes)	conductivity 56 mS cm^−1^ at 80 °C (1 wt%)	[67]
XL 3-(dimethylamino)-1-propylamine PSU	polydopamine-reduced GO (PDArGO)	Young’s modulus 1843 MPa (1.5 wt%)	[68]
TMA-PSU	XL reduced and functionalized GO (rGO)	conductivity 140 mS cm^−1^ at 80 °C (2 wt%)	[69]
TMA-PSU	rGO modified with pyrene-containing tertiary amines	conductivity 140 mS cm^−1^ 80 °C (2 wt%)	[70]
DABCO-PSU	C16 GO	19 × 10^5^ S min cm^−3^ selectivity for VRFB	[71]
BrPPO	(PEI)-GO	IEC 3.59 meq g^−1^	[72]
cellulose/DABCO-PPO	GO	conductivity 215 mS cm^−1^ at 80 °C	[73]
Im-PPO	1-(3-aminopropyl)-3-methylimidazolium bromine (IL-GO)	IEC decreased from 1.90 to 1.34 meq g^−1^ in 2 M NaOH, 80 °C, 480 h	[74]
Fumion^®^ FAA-3	graphene with surface area of 500 m^2^/g	conductivity 113 mS cm^−1^ at 80 °C	[75]
JAM-II-07 (Yanrun, China)	sulfonated rGO	area specific resistance 3.72 Ω cm^2^	[76]
sp-PBI	rGO	maximum power density 544 mW cm^−2^ at 90 °C	[77]
PBI	spin-coated GO	TS 50 MPa	[78]
PBI	MGO and NGO	conductivity 24 mS cm^−1^ (NGO) at 80 °C (1 wt%)	[79]
TMA-PAEK	rGO	Conductivity 115 mS cm^−1^ at 90 °C (1 wt%)	[80]
PPEK	QA-CDβ@GO	Young’s modulus 1243 MPa (10 wt%)	[81]
Im-PEEK	Im-GO	maximum power density 50 mW cm^−2^ at 50 °C (4 wt%)	[82]
DABCO-PAE	QGO	maximum power density 136 mW cm^−2^ at 70 °C (0.7 wt%)	[83]
perfluorinated AEM (I-PFSO_2_NH_2_-Cl)	IGNRs (GNRs grafted APTMS and MIMC)	conductivity 121 mS cm^−1^ at 80 °C (1 wt%)	[84]
XL-QSIBS	GO quaternized with: octadecylamine (GOA), octadecylamine + N,N-dimethyl-1,3-propanediamine (GOAN)	conductivity 19.5 mS cm^−1^ (GOAN 0.50 wt%) at 60 °C	[85]
QSIBS	poly (vinylbenzyl chloride) grafted graphene (GN-g-PVBC)	conductivity 18 mS cm^−1^ at 60 °C, storage modulus 418 MPa (0.55 wt%)	[86]
copolymer PMS/b-VIB/DPEBI	butylvinylimidazolium GO	conductivity 102 mS cm^−1^ at 100 °C	[87]
GO multilayer membranes	GO and GO_KOH_	water uptake GO_KOH_ 1099 wt%	[88]
TMA-PAES	g-C_3_N_4_	maximum power density 68 mW cm^−2^ at 60 °C (0.6 wt%)	[89]
TMA-PAEK	g-C_3_N_4_ nanosheets	conductivity 35 mS cm^−1^ at 80 °C (0.5 wt%)	[90]
N-methyl morpholine-PPO	f-BN	Yield stress 37 MPa (5 wt%)	[91]

**Table 4:** Type of polymer and of 3D filler in composite AEM with silica and silicate fillers.

Polymer	3D Filler	Remarks	Ref
XL TMA-PVDF	SiO_2_	conductivity 3 mS cm^−1^ at RT (2 wt%)	[95]
QCS	SiO_2_ coated PVDF grafted with trimethyl-3-(trimethoxysilyl) propyl ammonium chloride	conductivity 41 mS cm^−1^ at 80 °C (10.6 wt%)	[96]
XL PVA/3-(trimethylammonium) propyl-functionalized silica	SiO_2_	maximum power density 50 mW cm^−2^ at 60 °C (DEAFC)	[97]
3-(trimethyl ammonium)-PVA	3-(trimethyl ammonium) propyl-functionalized SiO_2_	storage modulus 172 MPa at 100 °C (20 wt%)	[98]
GTMAC-PVA	FS	conductivity 35 mS cm^−1^ at 60 °C (5 wt%)	[99]
CNC-PVA	SiO_2_	conductivity 65 mS cm^−1^ at 60 °C (40 wt%)	[100]
PVA and CS	AM-4, 4VP, AS4, UZAR-S3	conductivity 1 mS cm^−1^ at RT (4VP/CS:PVA)	[101]
TMA-PPO	SiO_2_	* U_H_* 0.041 m h^−1^, *S* 49	[102]
TMA-PES	functionalized SiO_2_ ASi-I, ASi-II, ASi-III	conductivity 46 mS cm^−1^ at 25 °C (3 wt%, ASi-II)	[103]
PPO	Im-SiO_2_	conductivity 105 m S cm^−1^ at 80 °C	[104]
TMA-PSU	TEA-SiO_2_ (QSBA)	OCV 0.86 V, power density 298 mW cm^−2^ (3 wt%)	[105]
TMA-PSU	TMA-SiO_2_ (QMSNs)	Young’s modulus 2250 MPa (20 wt%)	[106]
TEA-PSU	Im-mesoporous SiO_2_	maximum power density 278 mW cm^−2^ at 60 °C	[107]
TMA-PSU	modified montmorillonite	conductivity 47 mS cm^−1^ at 95 °C (5 wt%)	[108]
TMA-PSU	palygorskite	conductivity 93 mS cm^−1^ at 80 °C (0.5 wt%)	[109]
TMA-cardo-poly(etherketone) (QPEK-C)	N-(trimethoxysilylpropyl)-N,N,N-trimethylammonium	TS 26 MPa, elongation at break 32%	[110]

**Table 5:** Type of polymer and 3D filler in composite AEM with metal oxides and other inorganics.

Polymer	3D Filler	Remarks	Ref
GTMAC-PVA	Al_2_O_3_	conductivity 48 mS cm^−1^ at 70 °C	[111]
TMA-PSU	Al_2_O_3_	TS 31 MPa (4 wt%)	[112]
TMA-PSU	ZrO_2_	conductivity 15 mS cm^−1^ at RT	[113]
Im-PSU	ZrO_2_	conductivity 80 mS cm^−1^ at 50 °C	[114]
TMA-PAES	nano ZrO_2_	conductivity 48 mS cm^–1^ at 80 °C (10 wt%)	[115]
XL TMA-coPAES	nano ZrO_2_	Young’s modulus 492 MPa (10 wt%)	[116]
Perfluoro(phenyl 2,2:6,2-terpyridine); 2,2:6,2-terpyridine	ZrO(ClO_4_)_2_	IEC 0.76 meq g^−1^	[117]
TMA polystyrene-block-poly(ethylene-ran-butylene)-block-polystyrene (PSEBS)/TMA-PSU	SiO_2_, ZrO_2_, TiO_2_	maximum power density 75 mW cm^−2^ at 60 °C (7.5% TiO_2_)	[118]
TMA-PSU	TiO_2_	conductivity 13 mS cm^−1^ at 21 °C (10 wt% TiO_2_)	[119]
vinylbenzyl chloride-divinylbenzene copolymers	amorphous TiO_2_	conductivity 43 mS cm^−1^ at 30 °C	[120]
TMA-PPO/PSU	TiO_2_	maximum power density 118 mW cm^−2^ at 60 °C (2 wt%)	[121]
DABCO-PSU	TMP-TiO_2_ PMHS-TiO_2_	conductivity 39 mS cm^−1^ (PMHS-TiO_2_), 34 mS cm^−1^ (TMP-TiO_2_) at 25 °C in KOH 2M	[122]
TEA-PPO	1-methy-3-methylimidazolium-TiO_2_	Young’s modulus 921 MPa (TEA-PPO-1TiO_2_-15IL)	[123]
TEA-PPO	1-methy-3-methylimidazolium-TiO_2_	conductivity 52 mS cm^−1^ at 80 °C	[124]
TMA-PVA	TiO_2_	maximum power density 125 mW cm^−2^ at 35 °C	[125]
PVA	CaTiO_3_	conductivity 66 mS cm^−1^ at RT	[126]
PTFE	Sn_0.92_Sb_0.08_P_2_O_7_	maximum power density 147 mW cm^−2^ at 200 °C	[127]
CoOOH-PVA	CoSO_4_	maximum power density 144 mW cm^−2^ at 30 °C	[128]
TMA-PVA/CS	MoS_2_	TS 33 MPa	[129]
TMA-PPO/PSU	ZnO	maximum power density 69 mW cm^−2^ at RT	[130]
PBI	1-butyl-3-methyl imidazolium phosphotungstate	conductivity 76 mS cm^−1^ at 80 °C (20 wt%, PBI/(PWA-IL1:5))	[131]
poly vinyl benzyl trimethylammonium hydroxide	ZIF-8	BET 1045 to 600 m^2^ g^−1^	[133]
PVA	ZIF-8	conductivity 0.3 mS cm^−1^ at 60 °C	[134]
IL/PVA	IL@ZIF-8	conductivity 1.0 mS cm^−1^ at 60 °C (20 mol%)	[135]
PVA	ZIF-8	maximum power density 173 mW cm^−2^ at 60 °C (PVA/40.5% ZIF-8) (DMAFC)	[136]
BrPPO/PVA	MIL-101-Fe-NH_2_-F	conductivity 145 mS cm^−1^ at 80 °C	[137]
Im-PEEK	imidazolium MIL-101(Cr)	TS 35 MPa, (10 wt%)	[138]
Im-PSU	QCDs	Young’s modulus 1600 MPa	[139]
TMA-PSU	TMA-GQDs	conductivity 15 mS cm^−1^ at RT (0.1 wt%)	[140]

## Data Availability

The data presented in this study are available on request from the corresponding author.
